# Expanding the molecular spectrum of tenosynovial giant cell tumors

**DOI:** 10.3389/fonc.2022.1012527

**Published:** 2022-11-10

**Authors:** Thibault Gauduchon, Helene Vanacker, Daniel Pissaloux, Philippe Cassier, Armelle Dufresne, Marie Karanian, Alexandra Meurgey, Amine Bouhamama, François Gouin, Isabelle Ray-Coquard, Jean-Yves Blay, Franck Tirode, Mehdi Brahmi

**Affiliations:** ^1^ Département d’oncologie médicale, Centre de lutte contre le cancer Léon-Bérard, Lyon, France; ^2^ Centre de Recherche en Cancérologie de Lyon, INSERM U1052-CNRS5286, Lyon, France; ^3^ Université Claude Bernard Lyon 1, Faculté de médecine Lyon-Est, Lyon, France

**Keywords:** tenosynovial giant cell tumor (TGCT), CSF1, CSF1 fusion transcript, RNAseq analysis, expression profile, gene fusions

## Abstract

**Background:**

While great advances in clinical and pathological description of tenosynovial giant cell tumors (TGCT) have been made, TGCT molecular heterogeneity represents an ongoing challenge. The canonical oncogenic fusion *CSF1::COL6A3* is not systematically observed, suggesting that other oncogenic mechanisms are involved in tumorigenesis. This study aims to explore by RNA sequencing a retrospective series of tumors diagnosed as TGCT, in order to provide a better description of their molecular landscape and to correlate molecular features with clinical data.

**Methods:**

We analyzed clinicopathological data and performed whole-exome RNA sequencing on 41 TGCT samples.

**Results:**

RNAseq analysis showed significant higher CSF1 and CSF1-R expression than a control panel of 2642 solid tumors. RNA sequencing revealed fusion transcripts in 14 patients including 6 not involving CSF1 and some previously unreported fusions. Unsupervised clustering on the expression profiles issued from this series suggested two distinct subgroups: one composed of various molecular subtypes including *CSF1* and *FN1* rearranged samples and one composed of four tumors harboring an *HMGA2::NCOR2* fusion, suggesting distinct tumor entities. Overall, 15 patients received at least one systemic anti-CSF1R treatment and clinical improvement was observed in 11 patients, including patients from both clusters.

**Discussion:**

This study reported molecular heterogeneity in TGCT, contrasting with the clinical and pathological homogeneity and the ubiquitous high CSF1 and CSF1R expression levels. Whether molecular diversity may impact the efficacy of systemic treatments needs to be further investigated.

## Introduction

Tenosynovial giant cell tumors (TGCT) also called pigmented villonodular synovitis (PVNS) are rare tumors of unknown etiology that develop in the synovial tissue in young adults. TGCT are classified as localized type or diffuse type according to the extent of synovial involvement and the location (1, 2). Localized forms (l-TGCT) are often benign, non-destructive, well-circumscribed nodular in shape and mainly located distally in the limbs (3). On the other hand, diffuse types (d-TGCT) more often involve larger joints, especially the knee and are considered locally aggressive by the 2020 WHO Classification of Tumors of Soft Tissue and Bone (4).

Initially considered as an inflammatory reactive process, cytogenetic studies revealed recurrent translocations of 2q37 and 1p13 chromosomal loci involving *COL6A3* (encoding collagen type VI α3) and *CSF1* genes in a subpopulation of tumors (5) and showed that TGCT is a neoplastic process with a locally destructive clinical behaviour. The resulting fusion protein is cleaved and leads to uncontrolled CSF1 (colony-stimulating factor 1) secretion by tumor cells, which attracts non-neoplastic cells (ie, macrophages and monocytes) expressing the CSF1 receptor (CSF1R), *via* a paracrine effect (5).

The treatment of these tumors is mainly surgical but several systemic treatments have been investigated in case of local recurrence and/or locally advanced diseases (d-TGCT) (6–8). Imatinib, which inhibits CSF1-R among other group III receptor tyrosine kinases, has shown a good efficacy with 93% of disease control (9). Other more potent and selective tyrosine kinase inhibitors (TKI) targeting CSF1-R and anti-CSF1R monoclonal antibodies (mAbs) have been investigated (10–12).

More recent reports suggest that not all TGCT harbor a *CSF1::COL6A3* gene fusion and other rearrangements have been reported such as *CSF1::VCAM1*, *CSF1::FN1* and *CSF1::CDH1* fusions (13, 14). We herein describe clinicopathological and molecular features in a retrospective series of TGCT patients from the Centre Leon Berard, using RNA-sequencing.

## Methods

### Tumors samples

This monocentric retrospective translational research program was performed between November 2016 and November 2021 on tumor samples from an investigational cohort of 41 TGCT patients from the Centre Léon Bérard (Lyon, France). The inclusion criteria for the investigational cohort were: 1) histopathological diagnosis of TGCT centrally reviewed 2) sufficient available tumor material. All samples consisted of Formalin-Fixed Paraffin-Embedded (FFPE) tissues samples provided by the pathology department and collected by biopsy or surgical excision at initial diagnosis. The study was performed according to the French laws at the time of the initiation of the study and followed the principles of the Declaration of Helsinki. Oral consent was obtained after a detailed explanation to the patients or legal representatives for minors as reported in the patient’s medical record. As the study was not interventional, formal written consent was not required by French law. The Centre Léon Bérard Clinical Trial Review Committee (Local Review Board) reviewed and agreed with the study protocol and this consent procedure.

### Clinical review

Clinical follow-up was obtained from local medical records or provided by corresponding clinicians. Follow-up was reported from the date of diagnosis.

### Histopathological, RNA sequencing and gene expression

The diagnosis was confirmed by histopathological analysis and all cases of the series were reviewed by expert pathologists from the NETSARC+ network. Integrative whole-exome RNA sequencing and transcriptomic analysis was performed as previously described, with FFPE material from tumor biopsy or resection and before any systemic treatment (15).

### Statistical analysis

Patients and tumor characteristics were described using mean and range for continuous variables and percentages for categorical variables. Statistical analyzes and figures were performed with R software.

## Results

### Patients, clinical and pathological findings

**Figure 1:**
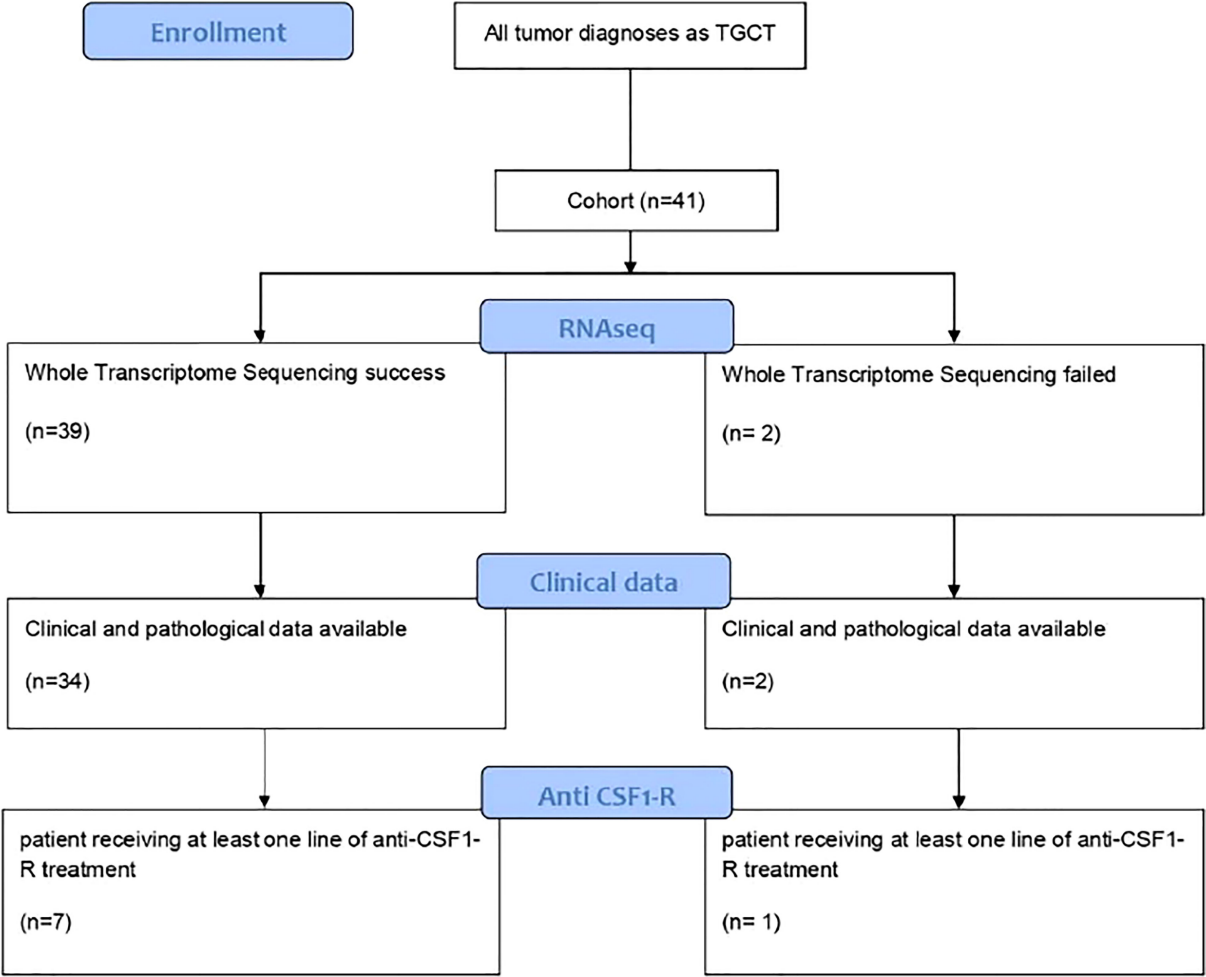
Cinsort diagram of patients included in the study.

This study identified 41 patients diagnosed with TGCT tumors (**Figure 1**). The clinical characteristics of the patients are presented in **Table 1**. The sex ratio was 1.4 and the mean age at diagnosis was 36.9 years (range 9-74 years), with a majority of d-TGCT (57%) as compared to l-TGCT. The knee joint was affected in 48% of cases and the ankle in 17% of cases. Other anatomical locations such as the feet, hands, hips and shoulders could also be affected. The main symptoms were pain (57%), swelling (53%) and limited range of motion (25%). Imaging identified lesions with different periarticular sizes and joint involvement (thickening of the synovium) in 71% of the cases, hemosiderin deposits visible in T2 gradient echo on MRI in 18% of the cases and lytic bone lesions in 18% of cases. Histology revealed histiocyte-like or epitheloid-like mononuclear cells in 100% of the cases, giant osteoclast-like plurinuclear cells in 91% of the cases, hemosiderin deposits with siderophages in 79% of the cases and foamy histiocytes in 58% of the cases. The lesion was more or less infiltrative or encapsulated depending on its localized or diffuse nature.

**Table 1:** Clinical characteristics of the investigational series of TCGT.

**N**	**41**
**Mean Age (years); range**	36.9; 9-74
**Sex ratio (M/F)**	1.4
**Location**	
Knee	17
Ankle	6
Hand	3
Foot	3
Other	6
N/A	6
**Type**	
Localized	12
Diffuse	16
N/A	13
**Main symptoms**	
Pain	16
Swelling	15
Limited range of motion	17
N/A	13
**Radiological lesions**	
Joint involvement	20
Hemosiderin deposits	5
Bone lysis	5
N/A	13
**Anatomopathology**	
Mononuclear cells	34
Giant plurinuclear cells	31
Hemosiderin deposits	27
Foam cells	20
N/A	7

N/A, not available.

### RNA sequencing analysis

**Figure 2:**
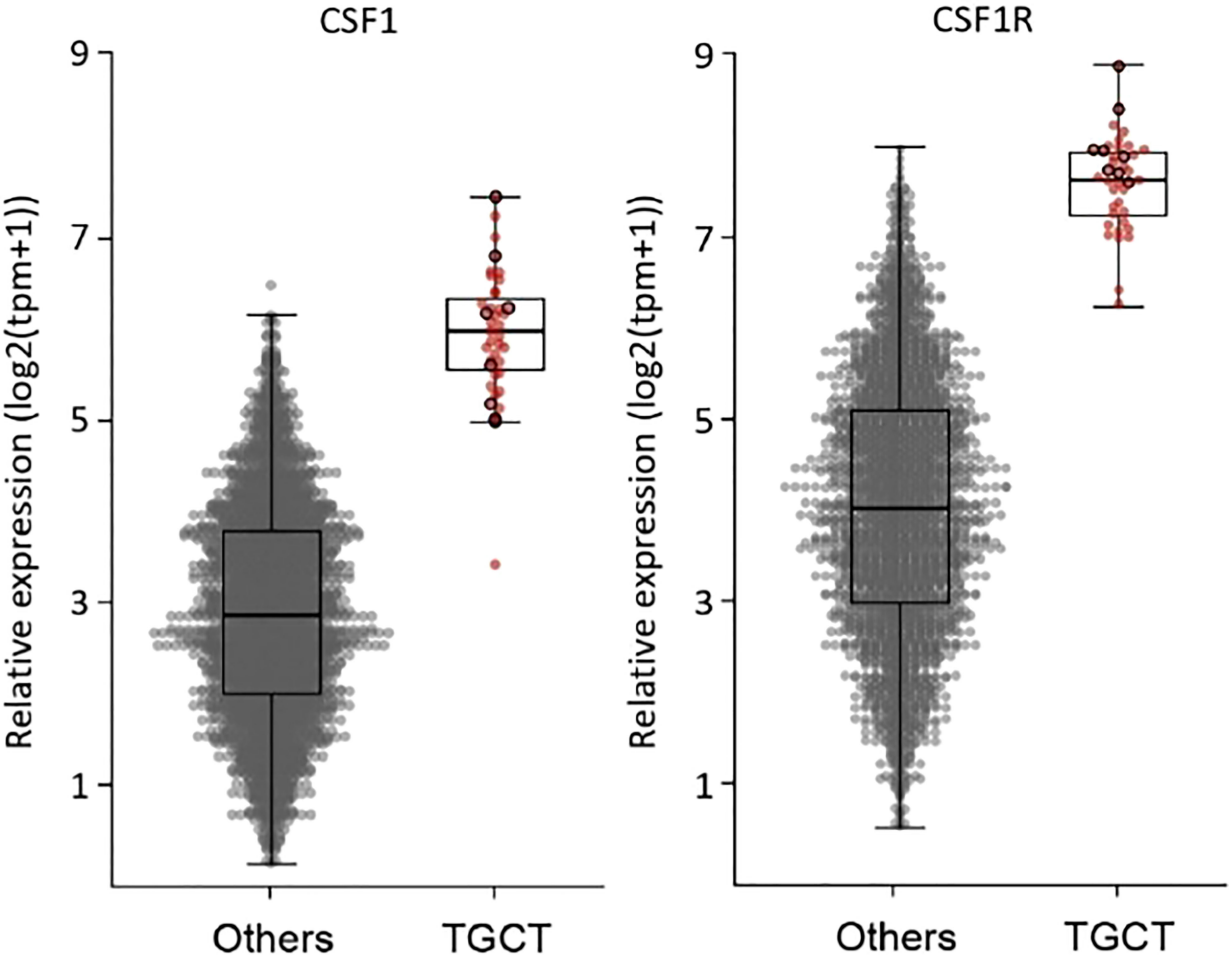
Overexpression of CSF1 and CSF1-R in all 39 tumors compared to a control panel of 2642 soild tumors by whole RNA sequencing.

**Figure 3:**
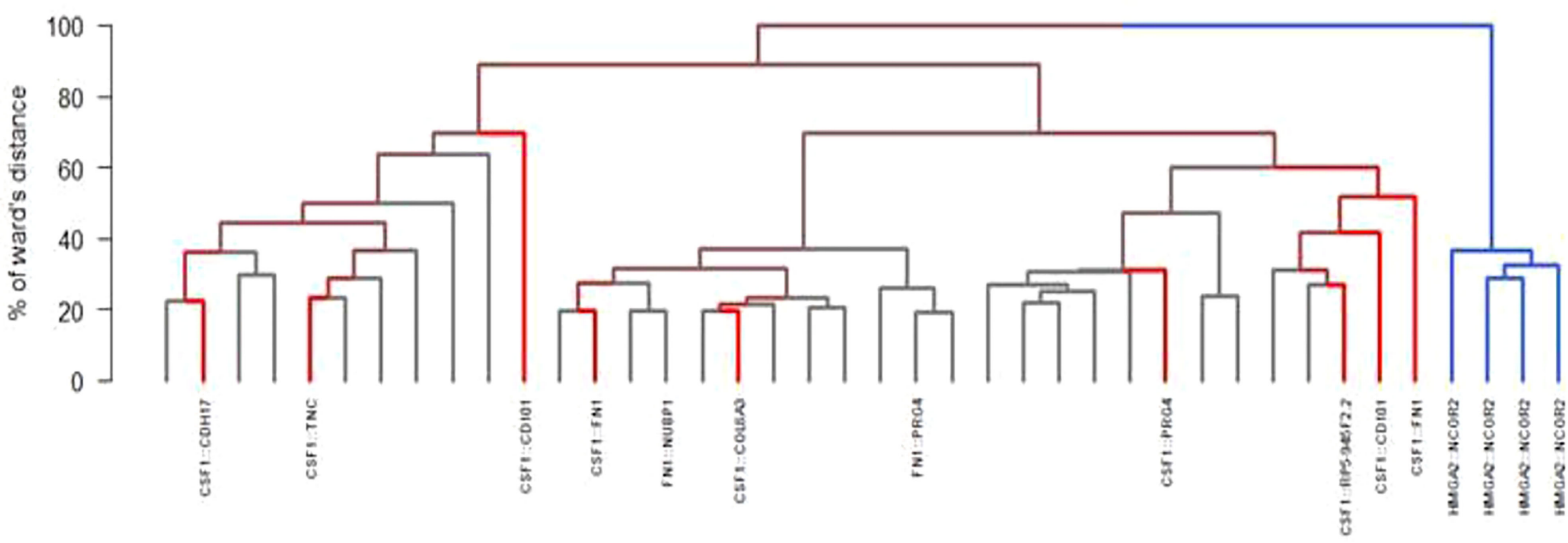
Different fusions found by RNA sequencing and hierchical ascending clustering of RNA sequencing (in blue: HMGA2::NCOR2 fusion; in red: non-HMGA2::NCOR2 fusion; in grey: no fusion detected).

All but two RNA-sequencing (RNA-seq) had successful quality controls (reads > 10M). Supervised analyses highlighted upregulation of *CSF1* and *CSF1-R* in all tumors (**Figure 2**) and this overexpression was significantly higher compared to a control panel of 2642 solid tumors with more than 240 different molecular subtypes (15). A chromosomal rearrangement was identified in 36% of the cases (*N*=14), with 8 of them involving *CSF1*. The fusion genes detected are shown in **Figure 3**. Considering the 8 cases with translocations involving *CSF1*, the fusion partners were respectively *FN1* (*N*=2), *CD101* (N=2). *COL6A3*, *PRG4*, *TNC* and *CDH17*. The other fusion transcripts detected were *HMGA2::NCOR2* (N=4), *FN1::PRG4* and *FN1::NUBP1*.

Unsupervised consensus hierarchical clustering divided the samples into two main groups/cluster. Cluster C1 (*N*=4) contained all the four *HMGA2::NCOR2* samples while cluster C2 (*N*=35) grouped various molecular subtypes, including *CSF1*-fused and *FN1*-fused samples. With a 3-dimensional principal component analysis, the 3-dimension explaining 7.5% of the total variation was due only to tumors with an HMGA2::NCOR2 fusion (**Supplementary Figure 1**). Significant difference between groups was observed with RNA-seq (**Supplementary Figure 2**). This difference is not likely to be related to microenvironment. (**Supplementary Figure 3**). The microenvironment was explored and the immune infiltrate was composed of dendritic cells, monocytes and non-M1 macrophages. This difference is also evidenced with RNA-seq results using Volcano plots showing different gene expression in C1 and C2 (**Supplementary Figure 4**). Mutations detected consisted all of variants of uncertain significance (*ATM*, *POLE*, *KMT2D*, *SMARCA4*, *TET2*, *GNAQ*, *BRCA2*, *PDGFRA/B* and *KIT*) except one case with a single nucleotide variant on the exon 13 of *CSF3R* (C.G1640A: P.W547x).

### Surgical and medical management

Among the 41 patients, 36 patients had detailed clinical management available. Among them, 11 patients (*N*=11/36, 31%) experienced initial surgery including five (*N* = 5/11, 45%) who had a recurrence after surgery. Mean time to relapse was 15.2 [3-48] months.

**Figure 4:**
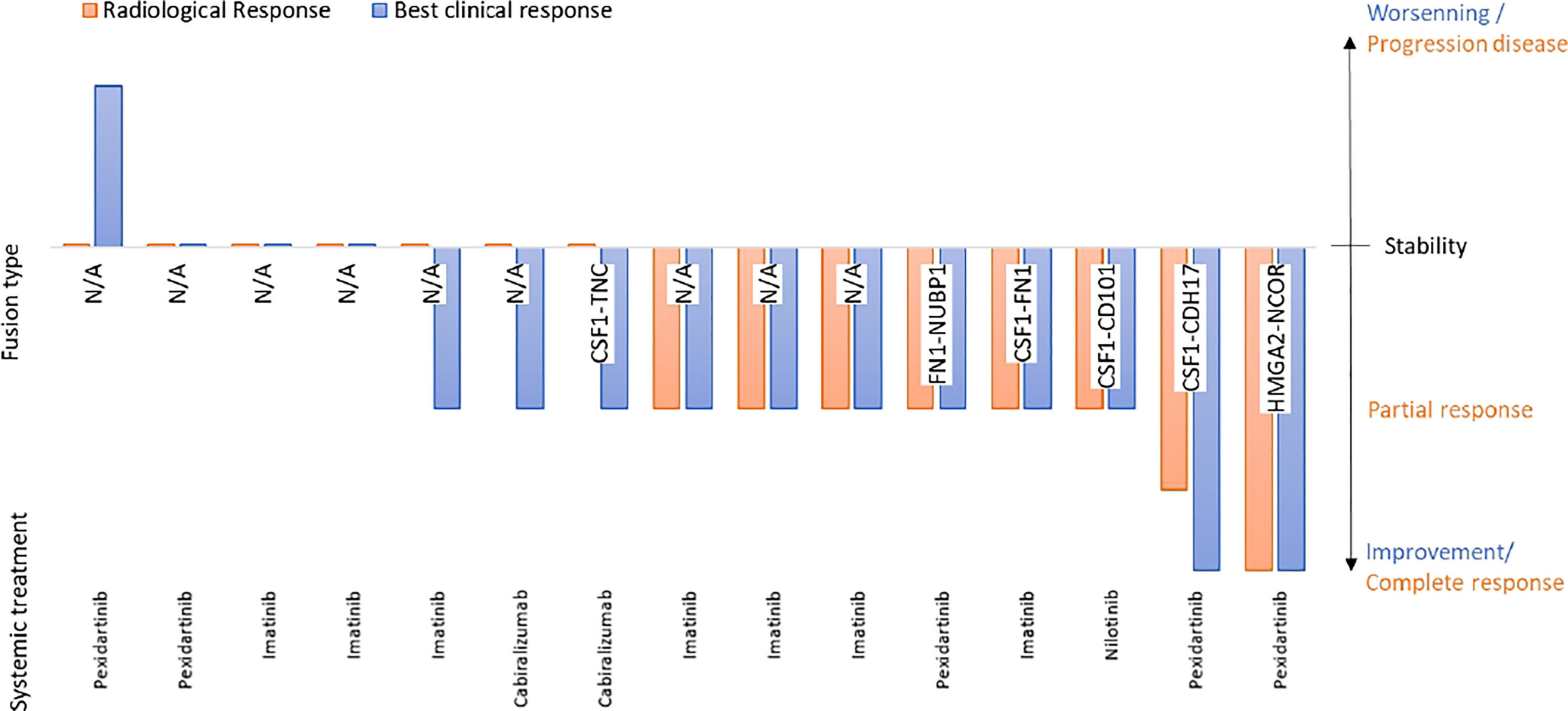
Correleation between gene fusion and medical treatment efficacy.

Fifteen patients received at least one systemic treatment targeting the CSF1/CSF1-R axis, eight received at least 2 lines of treatment and two patients received 3 lines of systemic treatment. The systemic therapies used were imatinib (*N*=11), pexidartinib (*N*=4), cabiralizumab (*N*=4), nilotinib (*N*=2) and emactuzumab (*N*=1). In the 15 patients treated with first line systemic treatment, two achieved a total improvement, 9 a partial improvement, three no improvement and 1 worsening of symptoms. Radiology revealed two complete response, 7 partial responses and 7 stable diseases. The mean duration of treatment was 10.8 months [1-54] and the mean relapse-free duration with this first line of systemic treatment was 25.9 months [2-64]. Treatments outcomes are detailed in **Figure 4**. Interestingly, a patient with an *HMGA2::NCOR2* gene fusion with an unresectable d-TGCT and achieved a CR with an anti-CSF1 TKI while a patient with a *FN1::NUBP1* fusion achieved a partial response after anti-CSF1 mAb therapy.

## Discussion

The present work analyzed a retrospective series of 41 TGCT including 39 with a contributive RNA-seq. All the tumors overexpressed *CSF1* and *CSF1-R*. The aberrant CSF1 expression in the neoplastic cells attracts non neoplastic cells, then forming a tumor mass through a landscape effect as previously proposed by West et al. in 2006 (5). Surprisingly, the historical translocation involving *CSF1* and *COL6A3* was detected in only one patient, despite a prevalence is estimated at 33% in the literature (16). A previous cohort of 39 TGCT reported no cases harboring the *COL6A3::CSF1* fusion using FISH and RNA-seq technics (14). Our results expand the molecular spectrum of this rare conjunctive tumor and a variety of genomic alterations is identified in this series. Interestingly, RNA-seq enabled us to suggest two groups combining gene fusion identification and expression profiling. The C2 group gathered TGCT harboring a fusion of *CSF1* and/or *FN1* while the C1 group is composed of tumors harboring an *HMGA2::NCOR2* fusion.

Overall, four samples harbored the *HMGA2::NCOR2* gene fusion, identified as the most represented fusion in our cohort. These fusions all showed a breakpoint was on *HMGA2* exon 3 and on *NCOR2* exon 14 or 15. The *HMGA2::NCOR2* fusion brings together a DNA-binding motif encoded by *HMGA2* exons 1–3 with *NCOR2* exons 16–47 encoding two repressor domains, a binding site for histone deacetylase 3 (HDAC3) and binding sites for several nuclear hormone receptors and transcription factors. Whether this fusion regulates the expression of *CSF1* is still unknown and the underlying molecular mechanisms remain elusive. Fusion genes involving *HMGA2* have also been detected in various tumor types such as lipomas leiomyomas, pleomorphic adenomas of the salivary gland, cervical polyps and hemangiopericytomas (17). This *HMGA2::NCOR2* fusion has recently been described in a series of 6 “giant cell tumors of soft tissue” (18) and in a series of 6 “osteoclastic giant cell-rich tumors of bone” (19). Unfortunately, expression of CSF1 was not detailed in these publications. In the four tumors harboring *HMGA2::NCOR2* fusion, two were identified in patients with a very atypical clinical presentation (soft tissue and temporal bone lesions). The distinctive clinical and genomic features strongly suggest that they belong to a distinct entity.

Regarding the translocations involving *CSF1*, the breakpoint was located on exon 5 in all but one case (one translocation on exon 6). These breakpoints may be responsible of *CSF1* overexpression in these tumors. Indeed, it is located downstream of exon 5, replaces or removes a long 3′UTR portion containing (AU)-rich elements known negative regulatory sequences of miRNA (14). We also identified some similarities with “non-COL6A3” CSF1 partners, usually expressed and secreted by synovial cells at high expression level and structurally linked to the extracellular matrix proteins (FN1, PRG4, TNC, CDH17) and therefore modulating the microenvironment through autocrine and/or paracrine activation. Indeed, the fibronectin binds to the surface of cells and to various compounds, including collagen, fibrin, heparin and actin. Fibronectins are involved in cell adhesion, cell motility, opsonization, wound healing and maintenance of cell shape. They are involved in osteoblast compaction through the fibronectin fibrillogenesis cell-mediated matrix assembly process, essential for osteoblast mineralization, and in the regulation of type I collagen deposition by osteoblasts. The *FN1::FGFR1* fusion gene is detected in phosphaturic mesenchymal tumors with paraneoplastic osteomalacia (20) and *FN1* has also been recently described as a novel fusion partner of *ALK* in an inflammatory myofibroblastic tumor (21). In addition, *FN1* is significantly overexpressed in TGCT such as the glycoprotein PRG4 which is secreted by synovial fibroblasts and superficial chondrocytes in the extracellular matrix and which regulates the proliferation of synoviocytes (22). *PRG4* has been identified as a fusion partner with *CSF1* but also with *FN1*, suggesting similar mechanisms.

While adequate surgical resection remains the treatment of choice for TGCT, d-TGCT is more difficult to resect and shows a higher rate of recurrence (up to 50%) (7, 23). Because TGCT is known to be associated with a *CSF1::COL6A3* fusion resulting in the overexpression of CSF1, systemic therapies targeting the CSF1/CSF1R axis (imatinib, nilotinib, emactuzumab, etc…) in patients with locally advanced and/or relapsed d-TGCT have been investigated and resulted in a very interesting clinical activity with acceptable toxicity (9–12). Nevertheless, the efficacy of these treatments is difficult to assess using conventional imaging criteria (RECIST) since multinodular disease with bone damage and joint pain limit the interpretation. The clinical response is therefore crucial and it underlines the need for relevant scales for functional, pain, or quality of life assessments (24, 25). The optimal treatment duration and the adequate schedule for neoadjuvant or adjuvant treatment also requires further investigation. Our cohort confirms the good overall response to the CSF1-R inhibitor, including in tumors harboring more uncommon fusions such as *HMGA2::NCOR2* (26) or *FN1::NUBP1*. Among the 11 patients who experienced initial surgery, five patients relapsed, which a mean time to relapse of 15.2 months. Of note, the mean relapse-free duration with a first line of systemic treatment was 25.9 months in our series.

To our knowledge, this study represents the largest series of RNA-sequenced TGCT. Altogether, TGCT represent a heterogeneous group of tumors in terms of genetics (27) including newly identified and some recurrent molecular variants. RNA-seq led to the reclassification of four “*HMGA2::NCOR2* giant cell tumors”. The transcriptional similarities between the CSF1 transcripts and newly identified transcripts suggest an analogous oncogenic activation pathway (28), illustrated by the efficacy of *CSF1* inhibitors in both situations.

## Data availability statement

The original contributions presented in the study are included in the article/**Supplementary Material**. Further inquiries can be directed to the corresponding author.

## Ethics statement

Ethical review and approval was not required for the study on human participants in accordance with the local legislation and institutional requirements. Written informed consent was not provided because As the study was not interventional, formal written consent was not required by French law. Ethical review and approval was not required for the animal study because as the study was not interventional, formal written consent was not required by French law.

## Author contributions

TG carried out the data collection and the drafting of the article. MB coordinated the scientific project. FT was responsible for the bioinformatics analyses. HV actively participated in the different stages of data analysis and writing of the article. All authors contributed to the article and approved the submitted version.

## Funding

Institut National du Cancer, EuroSARC (FP7 278742), LYRIC-INCA DGOS4664, Lyrican INCa_INSERM_DGOS_12563, LabEx DEVweCAN (ANR-10-LABX-0061), InterSARC grants, Fondation ARC pour la recherche sur le cancer, Grant Canopée from Ligue contre le Cancer (comité de l’Ain), Infosarcome, Association DAM’S.

## Conflict of interest

The authors declare that the research was conducted in the absence of any commercial or financial relationships that could be construed as a potential conflict of interest.

## Publisher’s note

All claims expressed in this article are solely those of the authors and do not necessarily represent those of their affiliated organizations, or those of the publisher, the editors and the reviewers. Any product that may be evaluated in this article, or claim that may be made by its manufacturer, is not guaranteed or endorsed by the publisher.

## References

[1] Mendenhall WM Mendenhall CM Reith JD Scarborough MT Gibbs CP Mendenhall NP. Pigmented villonodular synovitis. Am J Clin Oncol (2006) 29(6):548−50. doi: 10.1097/01.coc.0000239142.48188.f6 17148989

[2] Martin RC Osborne DL Edwards MJ Wrightson W McMasters KM. Giant cell tumor of tendon sheath, tenosynovial giant cell tumor, and pigmented villonodular synovitis: defining the presentation, surgical therapy and recurrence. Oncol Rep (2000) 7(2):413−9. doi: 10.3892/or.7.2.413 10671695

[3] Abdul-Karim FW el-Naggar AK Joyce MJ Makley JT Carter JR. Diffuse and localized tenosynovial giant cell tumor and pigmented villonodular synovitis: A clinicopathologic and flow cytometric DNA analysis. Hum Pathol (1992) 23(7):729−35. doi: 10.1016/0046-8177(92)90340-9 1319390

[4] WHO Classification of Tumours Editorial Board. Soft tissue and bone tumours Vol 3. 5th ed. International Agency for Research on Cancer) (2020) 607p

[5] West RB Rubin BP Miller MA Subramanian S Kaygusuz G Montgomery K. A landscape effect in tenosynovial giant-cell tumor from activation of CSF1 expression by a translocation in a minority of tumor cells. Proc Natl Acad Sci USA (2006) 103(3):690−5. doi: 10.1073/pnas.0507321103 16407111 PMC1325107

[6] Brahmi M Vinceneux A Cassier PA. Current systemic treatment options for tenosynovial giant cell Tumor/Pigmented villonodular synovitis: Targeting the CSF1/CSF1R axis. Curr Treat Options Oncol (2016) 17(2):10. doi: 10.1007/s11864-015-0385-x 26820289

[7] Mastboom MJL Palmerini E Verspoor FGM Rueten-Budde AJ Stacchiotti S Staals EL. Surgical outcomes of patients with diffuse-type tenosynovial giant-cell tumours: an international, retrospective, cohort study. Lancet Oncol (2019) 20(6):877−86. doi: 10.1016/S1470-2045(19)30100-7 31029509

[8] Healey JH Bernthal NM van de Sande M. Management of tenosynovial giant cell tumor: A neoplastic and inflammatory disease. J Am Acad Orthop Surg Glob Res Rev (2020) 4(11):e20.00028. doi: 10.5435/JAAOSGlobal-D-20-00028 PMC7643913 33156160

[9] Cassier PA Gelderblom H Stacchiotti S Thomas D Maki RG Kroep JR. Efficacy of imatinib mesylate for the treatment of locally advanced and/or metastatic tenosynovial giant cell tumor/pigmented villonodular synovitis. Cancer (2012) 118(6):1649−55. doi: 10.1002/cncr.26409 21823110

[10] Tap WD Gelderblom H Palmerini E Desai J Bauer S Blay JY. Pexidartinib versus placebo for advanced tenosynovial giant cell tumour (ENLIVEN): a randomised phase 3 trial. Lancet (2019) 394(10197):478−87. doi: 10.1016/S0140-6736(19)30764-0 31229240 PMC6860022

[11] Cassier PA Italiano A Gomez-Roca CA Le Tourneau C Toulmonde M Cannarile MA. CSF1R inhibition with emactuzumab in locally advanced diffuse-type tenosynovial giant cell tumours of the soft tissue: A dose-escalation and dose-expansion phase 1 study. Lancet Oncol (2015) 16(8):949−56. doi: 10.1016/S1470-2045(15)00132-1 26179200

[12] Gelderblom H Cropet C Chevreau C Boyle R Tattersall M Stacchiotti S. Nilotinib in locally advanced pigmented villonodular synovitis: a multicentre, open-label, single-arm, phase 2 trial. Lancet Oncol (2018) 19(5):639−48. doi: 10.1016/S1470-2045(18)30143-8 29571946

[13] Tsuda Y Hirata M Katayama K Motoi T Matsubara D Oda Y. Massively parallel sequencing of tenosynovial giant cell tumors reveals novel CSF1 fusion transcripts and novel somatic CBL mutations. Int J Cancer (2019) 145(12):3276−84. doi: 10.1002/ijc.32421 31107544

[14] Ho J Peters T Dickson BC Swanson D Fernandez A Frova-Seguin A. Detection of CSF1 rearrangements deleting the 3’ UTR in tenosynovial giant cell tumors. Genes Chromosomes Cancer (2020) 59(2):96−105. doi: 10.1002/gcc.22807 31469468

[15] Macagno N Pissaloux D de la Fouchardière A Karanian M Lantuejoul S Galateau Salle F. Wholistic approach: Transcriptomic analysis and beyond using archival material for molecular diagnosis. Genes Chromosomes Cancer (2022) 61(6):382–93. doi: 10.1002/gcc.23026 35080790

[16] Möller E Mandahl N Mertens F Panagopoulos I. Molecular identification of COL6A3-CSF1 fusion transcripts in tenosynovial giant cell tumors. Genes Chromosomes Cancer (2008) 47(1):21−5. doi: 10.1002/gcc.20501 17918257

[17] Dundr P Gregová M Hojný J Krkavcová E Michálková R Němejcová K. Uterine cellular leiomyomas are characterized by common HMGA2 aberrations, followed by chromosome 1p deletion and MED12 mutation: Morphological, molecular, and immunohistochemical study of 52 cases. Virchows Arch (2022) 480(2):281−91. doi: 10.1007/s00428-021-03217-z 34626221

[18] Agaimy A Michal M Stoehr R Ferrazzi F Fabian P Michal M. Recurrent novel HMGA2-NCOR2 fusions characterize a subset of keratin-positive giant cell-rich soft tissue tumors. Mod Pathol (2021) 34(8):1507–20. doi: 10.1038/s41379-021-00789-8 PMC8295036 33742141

[19] Panagopoulos I Andersen K Gorunova L Lund-Iversen M Lobmaier I Heim S. Recurrent fusion of the genes for high-mobility group AT-hook 2 (HMGA2) and nuclear receptor Co-repressor 2 (NCOR2) in osteoclastic giant cell-rich tumors of bone. Cancer Genomics Proteomics (2022) 19(2):163−77. doi: 10.21873/cgp.20312 35181586 PMC8865047

[20] Lee JC Su SY Changou CA Yang RS Tsai KS Collins MT. Characterization of FN1–FGFR1 and novel FN1–FGF1 fusion genes in a large series of phosphaturic mesenchymal tumors. Modern Pathol (2016) 29(11):1335−46. doi: 10.1038/modpathol.2016.137 PMC13417894 27443518

[21] Ren H Tan ZP Zhu X Crosby K Haack H Ren JM. Identification of anaplastic lymphoma kinase as a potential therapeutic target in ovarian cancer. Cancer Res (2012) 72(13):3312−23. doi: 10.1158/0008-5472.CAN-11-3931 22570254

[22] Alquraini A Jamal M Zhang L Schmidt T Jay GD Elsaid KA. The autocrine role of proteoglycan-4 (PRG4) in modulating osteoarthritic synoviocyte proliferation and expression of matrix degrading enzymes. Arthritis Res Ther (2017) 19(1):89. doi: 10.1186/s13075-017-1301-5 28482921 PMC5423025

[23] Palmerini E Staals EL Maki RG Pengo S Cioffi A Gambarotti M. Tenosynovial giant cell tumour/pigmented villonodular synovitis: outcome of 294 patients before the era of kinase inhibitors. Eur J Cancer (2015) 51(2):210−7. doi: 10.1016/j.ejca.2014.11.001 25465190

[24] Staals EL Ferrari S Donati DM Palmerini E. Diffuse-type tenosynovial giant cell tumour: Current treatment concepts and future perspectives. Eur J Cancer (2016) 63:34−40. doi: 10.1016/j.ejca.2016.04.022 27267143

[25] Bernthal NM Spierenburg G Healey JH Palmerini E Bauer S TOPP Study Group. The diffuse-type tenosynovial giant cell tumor (dt-TGCT) patient journey: a prospective multicenter study. Orphanet J Rare Dis (2021) 16(1):191. doi: 10.1186/s13023-021-01820-6 33926503 PMC8086070

[26] Brahmi M Alberti L Tirode F Karanian M Eberst L Pissaloux D. Complete response to CSF1R inhibitor in a translocation variant of teno-synovial giant cell tumor without genomic alteration of the CSF1 gene. Ann Oncol (2018) 29(6):1488−9. doi: 10.1093/annonc/mdy129 29668829

[27] Vougiouklakis T Shen G Feng X Hoda ST Jour G. Molecular profiling of atypical tenosynovial giant cell tumors reveals novel non-CSF1 fusions. Cancers (Basel) (2019) 12(1):E100. doi: 10.3390/cancers12010100 PMC7016751 31906059

[28] Tang Y Ma M Mi R Liu W Hou J Feng Y. Single-cell RNA-seq analysis reveals aberrant CSF1 expression in disease-causing synovial fibroblasts of pigmented villonodular synovitis ]. bioRxiv (2021):2021.09.29.462128. doi: 10.1101/2021.09.29.462128

