# Supplementary material for: Expanding the molecular spectrum of tenosynovial giant cell tumors
Source: Front Oncol. 2022 Nov 10;12:1012527. doi: 10.3389/fonc.2022.1012527 (PMC9691341; doi:10.3389/fonc.2022.1012527)

**Supplementary files**

**Supplementary Figure 1. Principal component analysis 3D of RNA sequencing:** with all genes on the left and only the 20% most variant gene on the right

**
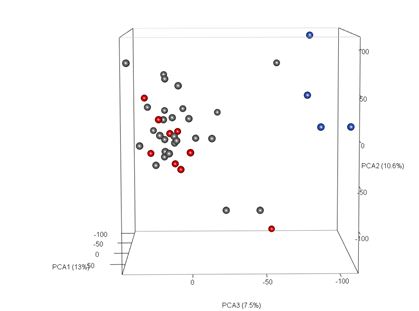

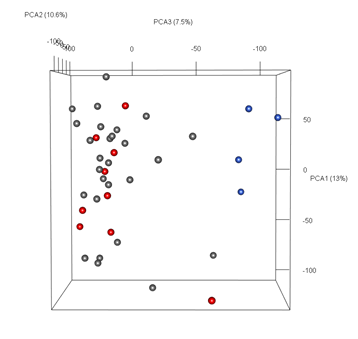
**

**Supplementary Figure 2. Difference in gene expression by RNAseq per cluster**


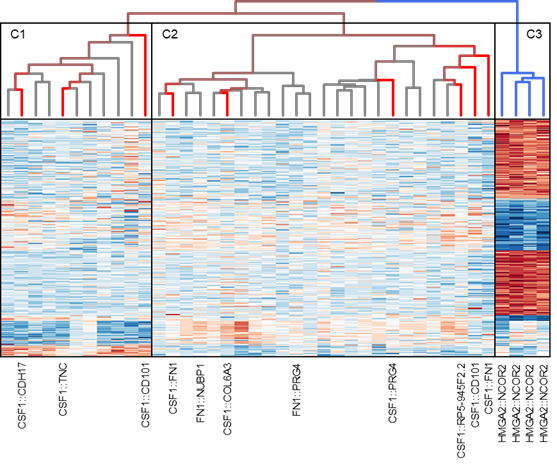


**Supplementary Figure 3. Difference in microenvironment expression by RNA seq per cluster**


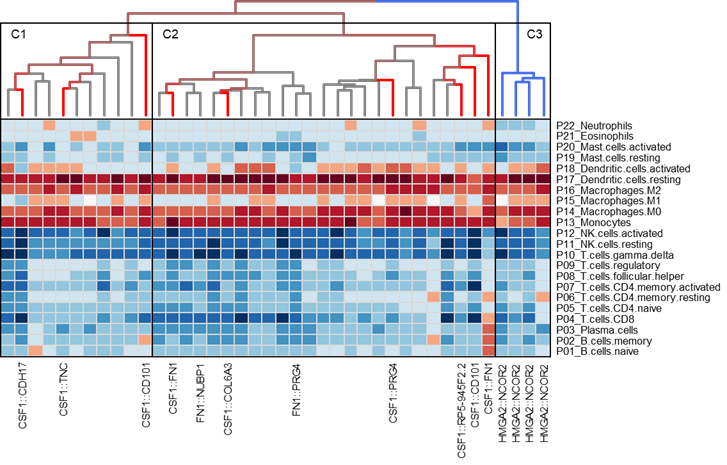


**Supplementary Figure 4. Volcano plot**

The most upregulated genes are towards the right, the most downregulated genes are towards the left, and the most statistically significant genes are towards the top.


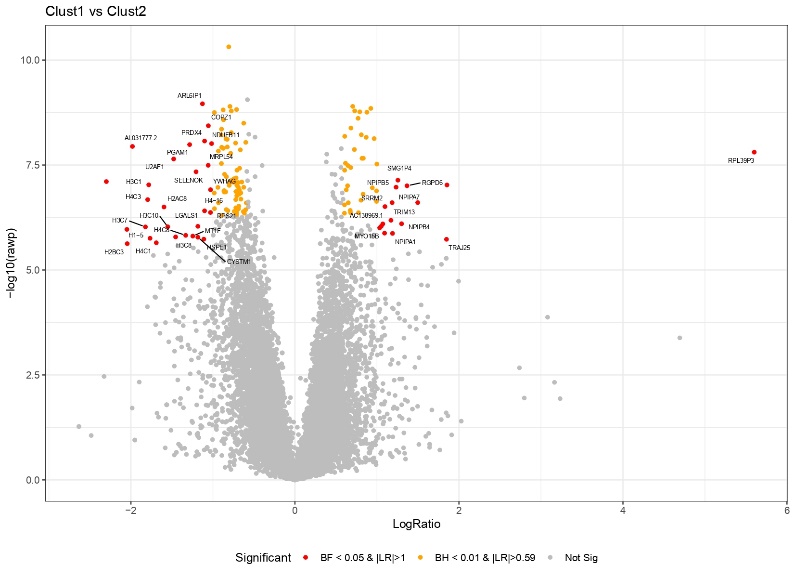

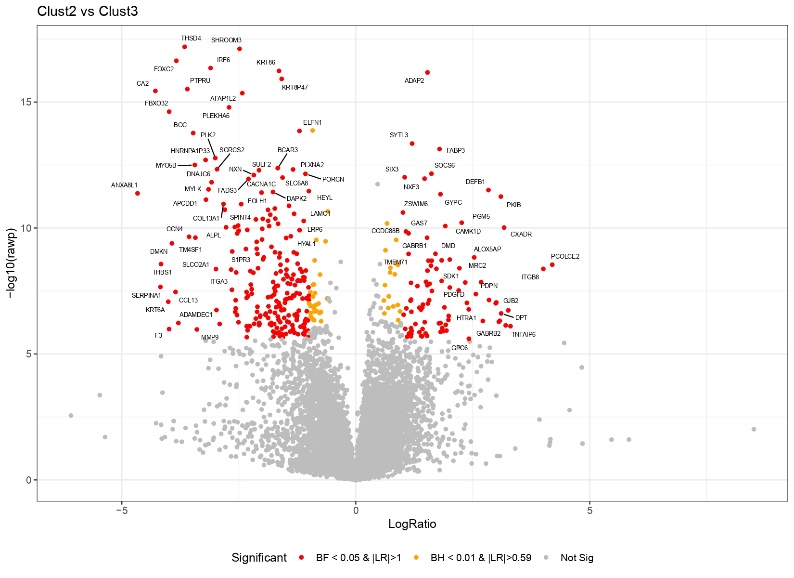

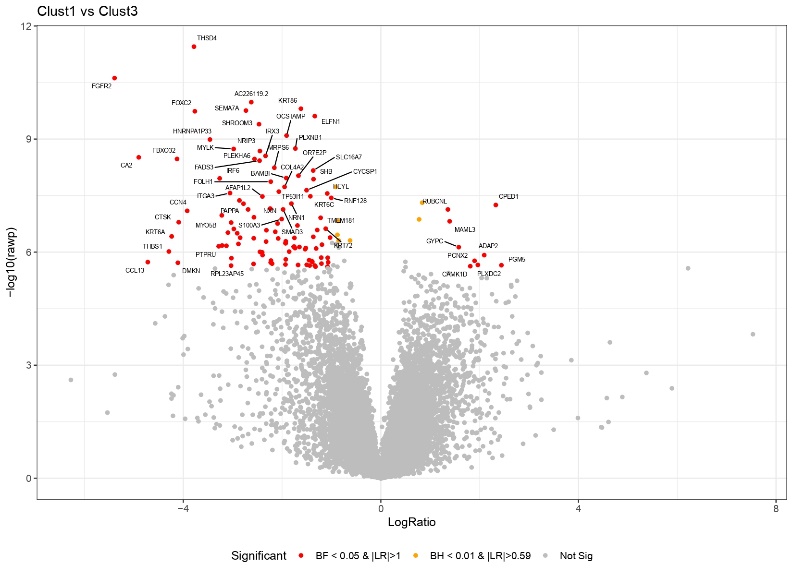

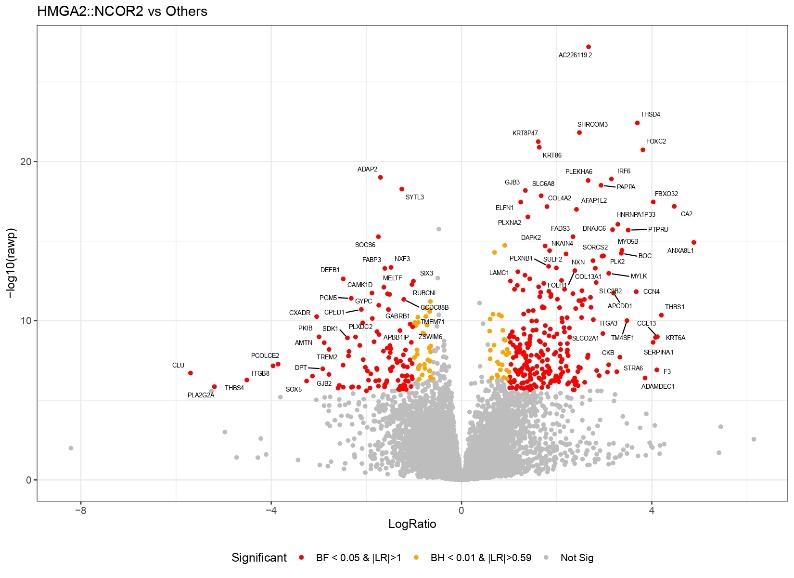

Supplement: Supplementary file 1 [file DataSheet_1.docx]
